# Cardiac glycoside-mediated turnover of Na, K-ATPases as a rational approach to reducing cell surface levels of the cellular prion protein

**DOI:** 10.1371/journal.pone.0270915

**Published:** 2022-07-01

**Authors:** Mohadeseh Mehrabian, Xinzhu Wang, Shehab Eid, Bei Qi Yan, Mark Grinberg, Murdock Siegner, Christopher Sackmann, Muhammad Sulman, Wenda Zhao, Declan Williams, Gerold Schmitt-Ulms

**Affiliations:** 1 Tanz Centre for Research in Neurodegenerative Diseases, University of Toronto, Krembil Discovery Centre, Toronto, Ontario, Canada; 2 Department of Laboratory Medicine & Pathobiology, University of Toronto, Toronto, Ontario, Canada; Ruhr University Bochum, GERMANY

## Abstract

It is widely anticipated that a reduction of brain levels of the cellular prion protein (PrP^C^) can prolong survival in a group of neurodegenerative diseases known as prion diseases. To date, efforts to decrease steady-state PrP^C^ levels by targeting this protein directly with small molecule drug-like compounds have largely been unsuccessful. Recently, we reported Na,K-ATPases to reside in immediate proximity to PrP^C^ in the brain, unlocking an opportunity for an indirect PrP^C^ targeting approach that capitalizes on the availability of potent cardiac glycosides (CGs). Here, we report that exposure of human co-cultures of neurons and astrocytes to non-toxic nanomolar levels of CGs causes profound reductions in PrP^C^ levels. The mechanism of action underpinning this outcome relies primarily on a subset of CGs engaging the ATP1A1 isoform, one of three α subunits of Na,K-ATPases expressed in brain cells. Upon CG docking to ATP1A1, the ligand receptor complex, and PrP^C^ along with it, is internalized by the cell. Subsequently, PrP^C^ is channeled to the lysosomal compartment where it is digested in a manner that can be rescued by silencing the cysteine protease cathepsin B. These data signify that the repurposing of CGs may be beneficial for the treatment of prion disorders.

## Introduction

Because PrP^C^ is essential for prion diseases to manifest [1], reducing its expression is widely considered one of the most promising avenues for their treatment. In contrast to approaches targeting the disease-associated misfolded conformer, PrP^Sc^, the ability to suppress the expression of PrP^C^ should confer protection in a manner that is prion strain-independent. There is robust evidence that reduced PrP^C^ levels due to heterozygous prion gene allele disruption does not cause major functional deficits in mice [2] or humans [3]. Moreover, complete prion gene -deficient mice are refractory to the disease [1] and exhibit, along with prion gene-deficient cattle [4] and goat [5], no overt pathological symptoms. Finally, the length of the symptom-free prion disease incubation period correlates inversely with the abundance of PrP^C^ [6, 7], an observation that can be conceptualized on the basis of PrP^C^ not only representing the substrate for conversion into PrP^Sc^ but also being critical for cellular toxicity [8]. Even when early prion disease symptoms have already taken hold, the lowering of PrP^C^ levels may partially reverse both the spongiform degeneration [9] and the neurophysiological dysfunction that contributes to the cognitive decline [10].

So far, efforts to identify PrP^C^-lowering drugs through screens of compound libraries have largely failed, with some of the best lead compounds either requiring relatively high concentrations to exert their effect or lacking favorable pharmacological characteristics for brain applications [11, 12]. Recent results from a study that targeted the stability of PrP^C^ transcripts with antisense oligonucleotides (ASOs) provided compelling proof-of-principle validation of the premise that lowering steady-state PrP^C^ levels can extend survival of prion-infected mice [7]. Adapting this approach to humans poses challenges, chiefly, the current necessity to inject ASOs periodically through the intrathecal, spinal cord route, because mRNA levels recover two months following bolus injections [13], and the formidable difficulty to deliver ASO to deep brain structures, a potential hurdle that is exacerbated in human adults due to their relatively large brain sizes [14].

We recently undertook systematic analyses of proteins that reside in proximity to PrP^C^ in four distinct mouse cell lines [15]. This work validated Ncam1 to be the most robust PrP^C^ neighbor in all paradigms we studied but also revealed Na,K-ATPases (NKAs) in proximity to PrP^C^ in three out of the four cell models tested. The terms neighbor and proximity are used in this context, in contrast to the more common term physical interactor, because we had stabilized proteins by time-controlled transcardiac perfusion crosslinking [16, 17], a technique that conceivably might stabilize next-neighbor relationships of proteins without a need for these proteins to be physical interactors. This finding spurred on further work, which led us to observe that NKAs are particularly prominent in immediate proximity to PrP^C^ in mouse brains [17], with only Ncam1 levels being more highly enriched in PrP^C^ co-immunoprecipitates obtained from mouse brains whose protein-protein interactions we had similarly stabilized by time-controlled transcardiac perfusion crosslinking. Subsequent work validated the interaction and documented partial co-localization of PrP^C^ and NKA in differentiated ReN VM cells, a human neural stem cell line [18]. These follow-on studies also revealed that the spatial proximity of PrP^C^ to NKAs influences the NKA-dependent potassium ion uptake into ReN VM cells. Although we currently lack information on the cell-type specificity and nuances of this PrP^C^ proximity to NKAs in differentiated ReN VM cells, we do know that PrP^C^ is expressed in all ReN VM cells regardless of whether they have acquired astrocytic or neuronal characteristics during their differentiation.

In the study before you, we exposed differentiated ReN VM cells to cardiac glycosides (CGs), a well-known class of NKA inhibitors, also known as cardiotonic steroids, and observed that the cells responded by reducing NKA steady-state levels. Remarkably, this response extended to PrP^C^, suggesting that this approach may offer a rational strategy for reducing its cell surface levels. We then explored the mechanism of action underlying the CG-dependent reduction of steady-state PrP^C^ levels from several angles. We show that this response can be elicited by several CGs in a concentration-dependent manner and is not restricted to the ReN VM cell model but can also be observed in T98G glioblastoma cells. Moreover, we document that not all paralogs of NKA α subunits respond equally to CG exposure and that the reduction in steady-state PrP^C^ levels depends on its degradation in an acidified endo/lysosomal compartment under participation of a specific cysteine protease. Finally, we show that the formation of a high-affinity ligand receptor complex between CGs and NKA α subunits is indispensable for the CG-dependent PrP^C^ reduction in human ReN VM cells.

## Results

### Sustained CG exposure of differentiated human neurons and astrocytes reduces PrP^C^ levels

**Fig 1:**
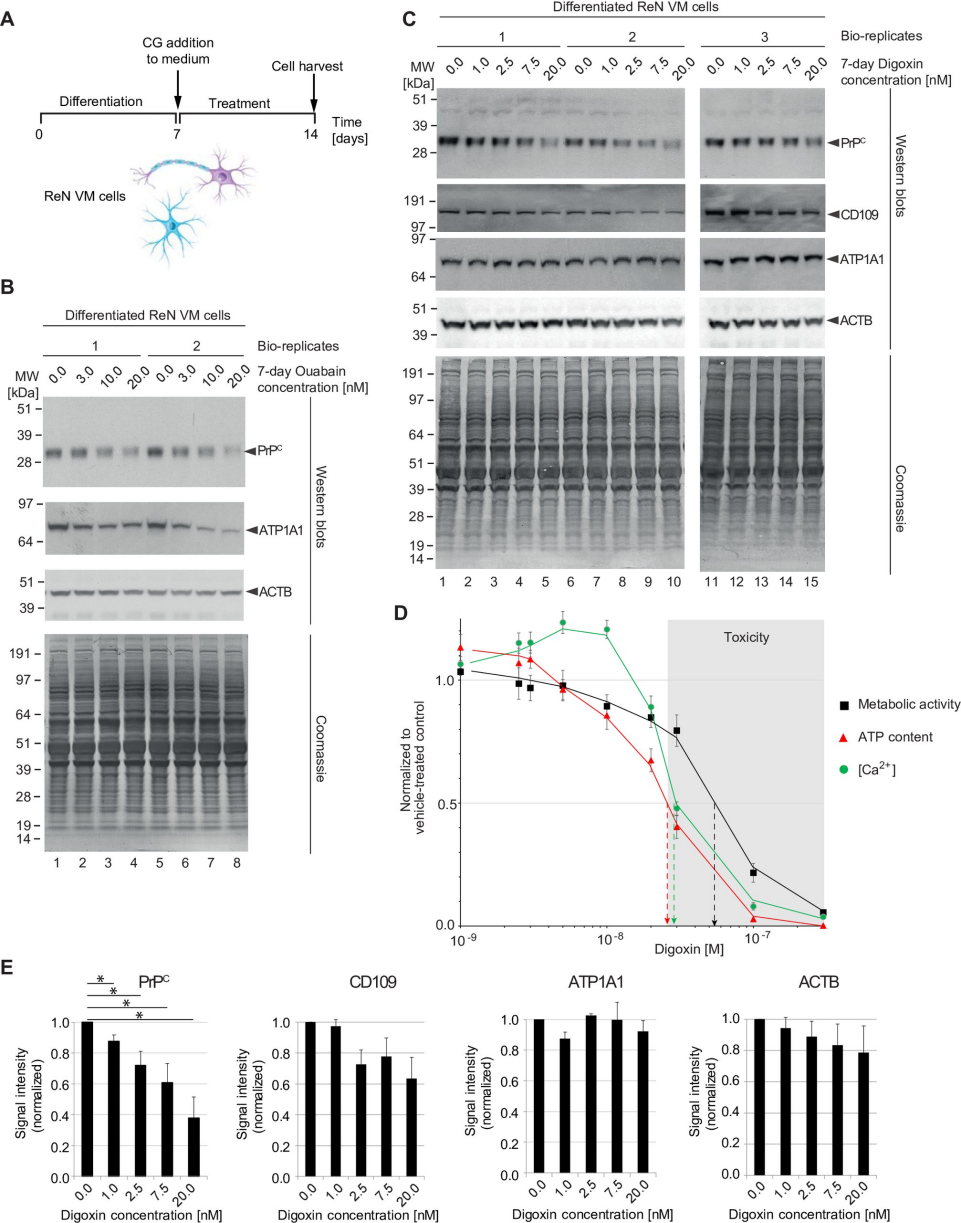
Prolonged non-toxic CG exposure of human ReN VM cells causes concentration-dependent reduction in NKA α subunit and PrP^C^ levels. (A) Workflow of *in vitro* ReN VM cell-based CG exposure analyses. (B) Exposure of differentiated ReN VM cells to ouabain at low nanomolar concentrations (up to 20 nM) caused a concentration-dependent reduction in PrP^C^ and ATP1A1 levels that was already apparent at 3 nM ouabain levels. Levels of actin or other proteins in the cell visualized by Coomassie staining were not similarly affected by ouabain. (C) Western blot documenting that extended exposure of differentiated neurons to low levels of digoxin (up to 20 nM) caused a dose-dependent reduction in PrP^C^ levels—and to a lesser extent steady-state protein levels of CD109 and actin—but did not affect the levels of ATP1A1 or many other cellular proteins, as evidenced by Coomassie staining. (D) 7-day ReN VM cell treatments with digoxin are toxic at concentrations exceeding 25 nM. Effects of a range of digoxin concentrations (0–500 nM) on cell viability (black squares), cellular ATP content (red triangles) and intracellular Ca^2+^ concentration ([Ca^2+^]_i_, green circles). Toxic concentrations of digoxin are shaded in grey. (E) Graphs depicting quantitation of steady-state levels of PrP^C^ and CD109 in the presence of non-toxic low nanomolar levels of digoxin.

To investigate the effect of CGs on NKA and PrP^C^ levels, ReN VM cells that had been differentiated for one week were exposed to a range of nanomolar concentrations of CGs or vehicle solution for a period of one week (Fig 1A). ReN VM cells were selected for these analyses because they offer a unique model for concomitantly studying the effect of CGs on human cells that have either acquired astrocytic or neuronal characteristics, with their neurons previously reported to sustain action potentials following differentiation [18]. Western blot analyses of total cellular extracts obtained from these cells documented a ouabain concentration-dependent depletion of PrP^C^ at ouabain levels of 3 nM to 20 nM, i.e., near the previously determined K_D_ for this pump [19]. The reduction in steady-state PrP^C^ levels followed the depletion of the ATP1A1 isoform, the most broadly expressed NKA α subunit encoded in the human genome (reviewed in [20]). At the concentrations tested, ouabain did not affect bulk protein levels, which were monitored by Coomassie staining (Fig 1B).

Whereas ouabain is primarily used in experimental research, the CGs known as digoxin and digitoxin are still used in the clinic today. In part, this distinction reflects the observation that ouabain dissociates from NKAs after less than two hours [21], whereas digoxin is capable of a more sustained NKA inhibition due to its almost tenfold slower dissociation [22]. To explore if administration of digoxin can similarly reduce PrP^C^ levels at low nanomolar concentrations, we next repeated the analyses with digoxin added to the ReN VM cell culture medium at levels that ranged from 1 nM to 20 nM. Data collected from three biological replicates for each digoxin concentration tested (1, 2.5, 7.5 and 20 nM) demonstrated that digoxin exposure again led to no discernible changes in ReN VM cell bulk protein levels. Remarkably, and in contrast to the results we had obtained with ouabain, digoxin treatment did not significantly affect steady-state ATP1A1 levels, yet also caused a concentration-dependent reduction in PrP^C^ levels at concentrations as low as 1 nM digoxin, a level achieved in human serum samples in the clinic [23]. The levels of CD109, another GPI-anchored protein that we had previously reported to reside in proximity to PrP^C^ in certain cell types [15], were also reduced in digoxin-exposed cells, albeit to a lesser extent than PrP^C^ (Fig 1C).

To determine the cellular health of ReN VM cells treated with digoxin, we assayed metabolic activity (calcein-AM assay), levels of intracellular ATP content (CellTiter-Glo assay), and intracellular calcium levels (Fluo-4 AM assay) of ReN VM cells cultured in the presence of 1 nM to 300 nM digoxin using a microplate reader (Fig 1D). These analyses revealed that the levels of cellular calcium initially increased by approximately 20% as intracellular ATP levels and metabolic health began to decline in the presence of low nanomolar concentrations of digoxin. In ReN VM cells treated with digoxin levels that exceeded 10 nM all three measures of cellular health dropped relative to mock-treated control cells. Cellular toxicity—operationally defined as a drop of ATP levels to 50% of those seen in mock-treated control cells—was apparent in cells exposed to digoxin levels exceeding 25 nM. Half maximum intracellular calcium levels were seen with digoxin levels in the cell medium at 30 nM, and metabolic activity dropped to 50% at around 50 nM digoxin levels.

Quantitative image analyses of western blot signals revealed steady-state PrP^C^ levels in the presence of 20 nM digoxin to be reduced to approximately 40% of levels observed in vehicle treated cells (Fig 1E). Steady-state protein levels of CD109 were reduced approximately half as much as PrP^C^ levels under these conditions to 70% of signal intensities observed in vehicle-treated cells. These results established that a significant reduction in steady-state PrP^C^ levels can be elicited in a concentration-dependent manner by more than one CG and extends to some degree to other nearby proteins. The differences in the degree to which ouabain and digoxin affected ATP1A1 protein levels also suggested that it might be worthwhile to explore how other NKA α subunits are impacted by CG exposure.

### CG-dependent changes to PrP^C^ levels are more closely associated with ATP1A1 than with ATP1A2 protein levels

The human genome codes for four NKA α subunits and three β subunits that can assemble into active NKAs, and most cell types express restricted combinations of these subunits [24–27]. For instance, whereas neurons are known to express ATP1A1 and ATP1A3 α subunits, astrocytes express primarily ATP1A2. Moreover, CGs exhibit distinct binding profiles toward NKAs composed of specific subunits [28, 29].

**Fig 2:**
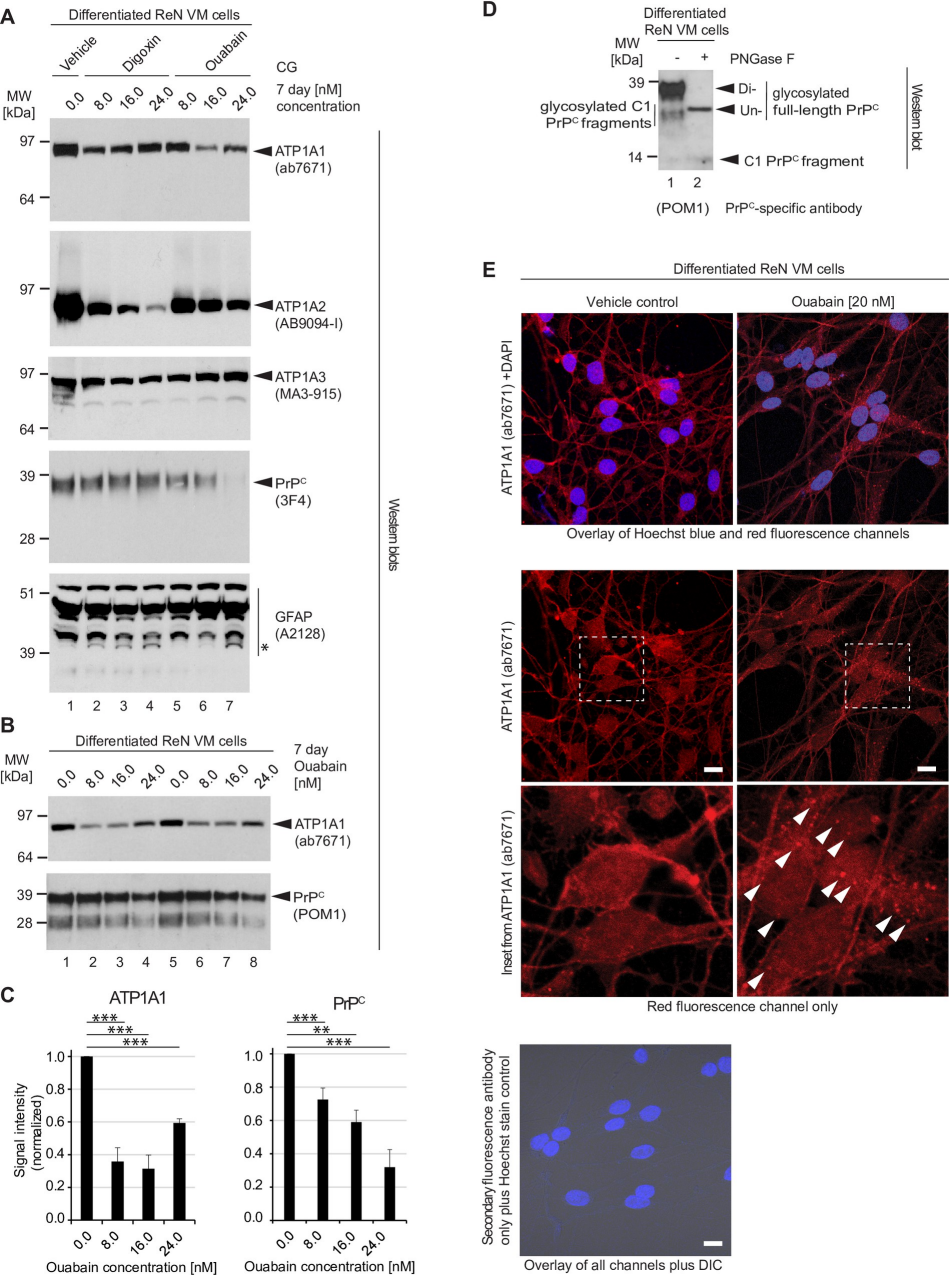
Digoxin and ouabain affect steady-state levels of NKA α subunits differently, and CG-dependent changes to PrP^C^ levels are more closely associated with ATP1A1 than ATP1A2 protein levels. (A) Digoxin exerts a lesser effect on steady-state ATP1A1 than ATP1A2 levels. The opposite is observed for ouabain exposed cells. Cellular extracts of 7-day differentiated, then 7-day CG-treated ReN VM cells were adjusted in their protein concentration and analyzed by western blotting with probes directed against NKA α subunits, PrP^C^ and GFAP. Note that 24 nM levels of digoxin (Lane 4) diminished ATP1A2 protein levels but had relatively little effect on ATP1A1 protein levels. In contrast, 16 nM ouabain dramatically depletes ATP1A1 protein levels, with little changes to ATP1A2 protein levels (Lane 6). Levels of 3F4-reactive PrP^C^ signals were more profoundly reduced in cells exposed to ouabain than digoxin (compare Lanes 4 and 7). GFAP levels were mostly unchanged. However, consistent with results we had reported before [17], a GFAP-antibody reactive band (see asterisk), migrating with an apparent molecular weight of ~39 kDa, became more pronounced in CG exposed cells. (B) Whereas steady-state levels of ATP1A1 recover at borderline toxic ouabain concentration of 24 nM, steady-state levels of PrP^C^ remain inversely correlated with ouabain levels within the window of ouabain concentrations tested in this experiment. Note that this western blot was probed with the PrP^C^-reactive antibody POM1, which detects both full-length and endoproteolytically digested C1 fragments of PrP^C^. (C) Quantitation of ATP1A1 and PrP^C^ western blot signals following 7-day ouabain treatment. (D) Following 7 days of differentiation, ReN VM cell cultures primarily express diglycosylated full-length PrP^C^. Cellular extracts of ReN VM cells were analyzed without or with prior PNGase F digestion and western blot detection of POM1-reactive signals. (E) Ouabain administration elicits internalization of ATP1A1 in 7-day differentiated ReN VM cells. Under basal conditions, ATP1A1 localizes primarily to the plasma membrane. Following treatment with 20 nM ouabain, ATP1A1 is increasingly internalized by cells into punctate structures (white arrowheads). Scale bar = 10 μm.

Because the digoxin-dependent reduction in PrP^C^ levels was not accompanied by a pronounced reduction in steady-state ATP1A1 protein levels, we hypothesized that this result may reflect previously reported differences in the affinity of ouabain versus digoxin toward specific NKA α subunits, with ouabain reported to bind preferentially to ATP1A1 and digoxin to ATP1A2 [30]. We therefore exposed ReN VM cells to digoxin versus ouabain using the same differentiation and treatment protocol as before (Fig 1A), then compared the levels of the three NKA α subunits ATP1A1, ATP1A2 and ATP1A3 by western blot analyses. Our observations corroborated the concept of cells responding to CG exposure in a manner that reflects both the distinct binding properties of individual CGs and the NKA expression profile of the target cell. More specifically, whereas even 24 nM concentrations of digoxin caused only a minor reduction in ATP1A1 levels, it profoundly reduced levels of ATP1A2 (Fig 2A). In converse, as little as 16 nM of ouabain caused a strong reduction in the steady-state ATP1A1 levels yet at this ouabain concentration ATP1A2 levels were only slightly reduced. As the ouabain concentration was increased to 24 nM, ATP1A1 levels were again stronger (Fig 2B and 2C). The impact of either drug on ATP1A3 levels was more subtle, with digoxin exposure leading to a minor concentration-dependent decrease and ouabain to a minor increase in the steady-state protein levels of this NKA α subunit. Both CGs reduced PrP^C^ protein levels but the side-by-side comparison revealed ouabain to be more potent in this regard. Because these experiments were undertaken with differentiated ReN VM cells, we wondered if the presence of digoxin or ouabain had altered the balance and differentiation state of this co-culture paradigm comprising astrocytic and neuronal cell subpopulations. Using an antibody directed against GFAP as a marker for astrocytes, we observed that GFAP levels mostly did not change in response to CG treatment, indicating that the dramatic reduction in ATP1A2 protein levels did not merely reflect a selective decimation of astrocytes (Fig 2B). These analyses did, however, further validate our previously reported observation that the treatment with either CG was associated with the appearance of a low molecular weight GFAP antibody-reactive signal of around 39 kDa that we had interpreted as an endoproteolytic calpain cleavage product [17].

These experiments indicated that shifts in the steady-state levels of cellular NKA subunits in response to digoxin or ouabain reflect indeed their preferential binding to ATP1A2 and ATP1A1, respectively, and that PrP^C^ protein levels are more closely associated with protein levels of ATP1A1 than ATP1A2. When we characterized PrP^C^ isoforms expressed in ReN VM cells with the PrP^C^-reactive antibody POM1 (with and without prior PNGase F digestion), it became apparent that the majority of PrP^C^ in this paradigm consists of diglycosylated full-length PrP^C^ (Fig 2D), which primarily resides at the cell surface.

It has previously been reported that ouabain binding can lead to the removal of NKA complexes from the cell surface through cellular internalization [31]. To begin to explore if this phenomenon also applies to one-week differentiated ReN VM cells, we made use of immunocytochemical analyses to learn if ATP1A1 signals become internalized in cells exposed to 20 nM ouabain (Fig 2E). As expected, a subset of ATP1A1 antibody-reactive signals became associated with punctate structures within ouabain-exposed but not vehicle-treated cells. This result corroborated the concept of a CG-dependent internalization but did not reveal the cellular internalization and degradation program responsible.

### The CG-induced reduction in PrP^C^ levels involves lysosomal degradation by a cysteine protease

**Fig 3:**
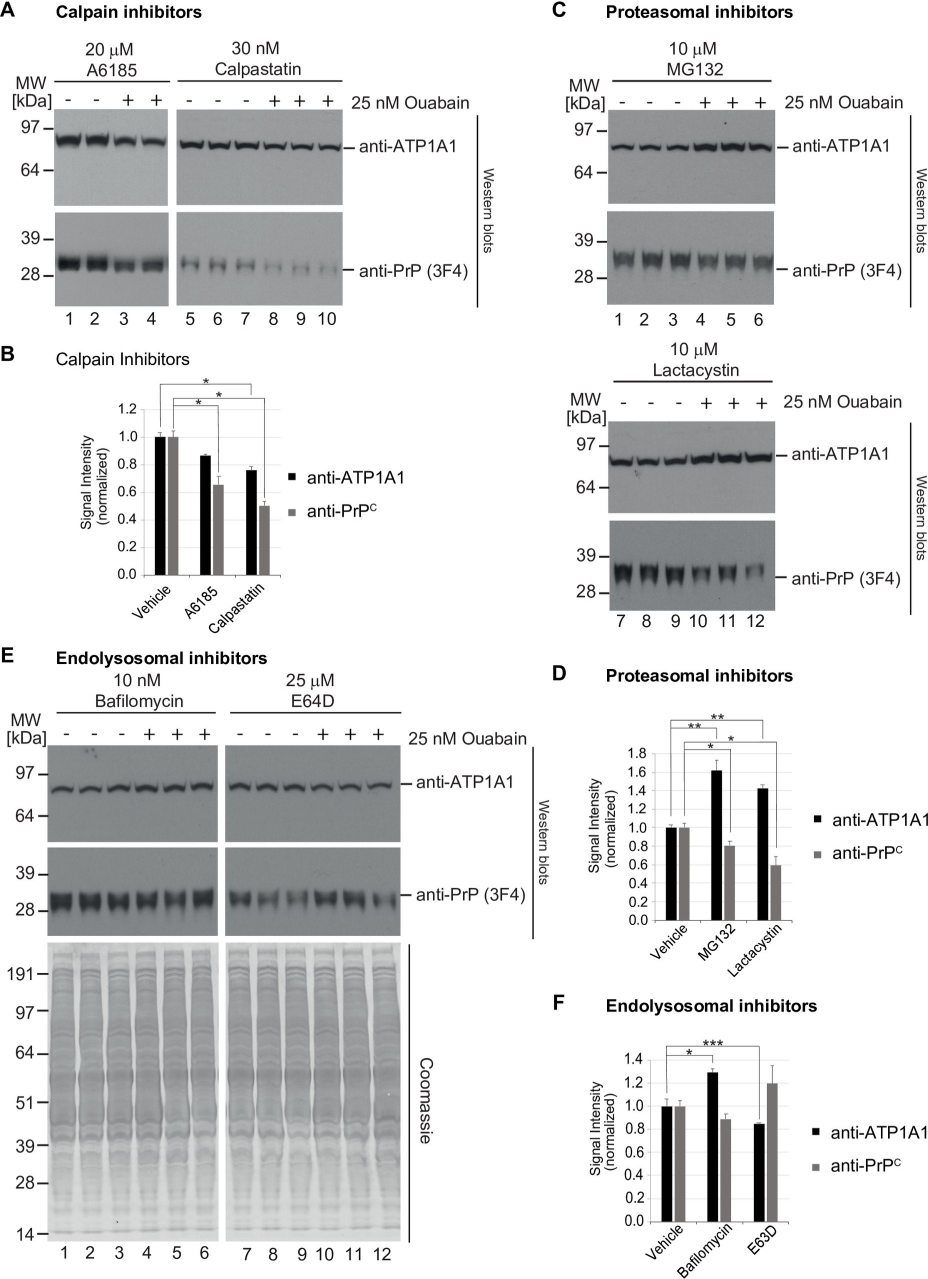
CG-dependent reduction of PrP^C^ levels relies predominantly on endo/lysosomal degradation pathway. (A,B) Inhibition of calpain proteases with A6185 or calpastatin did not rescue the ouabain-dependent reduction in steady-state ATP1A1 or PrP^C^ levels in ReN VM cells. (C,D) Proteasomal inhibition with MG132 or lactacystin caused an increase in steady-state ATP1A1 levels yet did not rescue the reduction in PrP^C^ levels that is observed in ouabain-treated cells. (E,F) In contrast, inhibition of endo/lysosomal degradation pathways with bafilomycin A1 or E64D blocked the ouabain-dependent PrP^C^ reduction. Quantitative graphs in this figure depict the means of signal intensities and their standard deviations. Asterisks reflect significance thresholds with one asterisk shown if t-tests returned *p*<0.05 and two asterisks indicating *p*<0.005.

Steady-state levels of PrP^C^ are known to be modulated to varying degrees by calpain-dependent proteolysis [32], as well as proteasomal [33, 34], and endo/lysosomal degradation [35, 36], i.e., each of the three dominant protein degradation systems in eukaryotic cells. To explore if any of these systems plays a role in the CG-dependent reduction in steady-state PrP^C^ levels, we next repeated the ouabain treatment of ReN VM cells in the presence or absence of well-established inhibitors of these degradation pathways. Because the selectivity of available inhibitors is crude, two distinct inhibitors were tested separately for each of the three degradation pathways. The design of this experiment was based on the hypothesis that one of the three degradation pathways may by itself account for the CG-induced reduction in PrP^C^ levels. Consequently, ReN VM cells were again exposed to mock treatment or to 25 nM ouabain. Next, it was observed whether the concomitant presence of an inhibitor of one of the three degradation system can block the expected reduction in PrP^C^ levels. When ReN VM cells were tested in this manner, the presence of two calpain inhibitors (A6185 or calpastatin) did neither prevent the CG-induced reduction in ATP1A1 levels nor the reduction in PrP^C^ protein levels (Fig 3A and 3B). The presence of proteasomal inhibitors (MG132 or lactacystin) also failed to block the CG-dependent reduction in PrP^C^ protein levels but, intriguingly, caused an increase in ATP1A1 steady-state protein levels (Fig 3C and 3D). Finally, and in contrast to the aforementioned inhibitors, a blockade of endo/lysosomal proteolysis, either through inhibiting acidification of endo/lysosomal compartments (bafilomycin) or through the addition of a cysteine protease inhibitor, which irreversibly modifies the active thiol group present in cysteine proteases (E-64), rescued the CG-induced steady-state level reductions of both ATP1A1 and PrP^C^ (Fig 3E and 3F). Taken together, these experiments indicated that the endo/lysosomal degradation pathway is critical for the CG-dependent reduction in steady-state PrP^C^ protein levels.

### Cathepsin B contributes to the CG-induced endo/lysosomal reduction of PrP^C^ levels

We hypothesized that members of the cathepsin family are likely candidates for mediating the CG-induced PrP^C^ reductions because these proteases dominate endo/lysosomal protein degradation activities in the brain [37]. Moreover, a subset of cathepsins had previously been shown to be upregulated in prion-infected mouse brains [38] or cells [39], had been reported to partially cleave PrP^C^ *in vitro* [40], remove its GPI-anchor [41], or promote its degradation [42]. In determining which of the cathepsins are plausible candidates, we noted that a recent manuscript reporting on the levels of expression of individual cathepsins in the human brain [43] mostly corroborated Human Protein Atlas (HPA) cathepsin protein expression data, and therefore decided to shortlist all cathepsins for testing that the HPA reported to be expressed in the brain at moderate to high levels, ignoring others which were reported to exhibit no or very low expression in this tissue (Table 1).

**Table 1:** Summary of information that guided the shortlisting of cathepsins for determining their possible involvement in the CG-dependent reduction of PrP^C^ levels.

Name (alias)	Gene	Type	HPA brain RNA	HPA brain protein	Increased in prion-infected mouse brains	Increased in prion-infected N2a cells	Cleaves PrP^C^ *in vitro*	Removes GPI anchor of PrP^C^ *in vitro*	Promotes PrP^Sc^ degradation in GT1 cells
Cathepsin A	*CTSA*	Serine	low	high					
Cathepsin B	*CTSB*	Cysteine	low	high	x	x	x		x
Cathepsin C	*CTSC*	Cysteine	very low	low	x				
Cathepsin D	*CTSD*	Aspartyl	low	high	x			x	
Cathepsin E	*CTSE*	Aspartyl	nil/nil	nil/nil					
Cathepsin F	*CTSF*	Cysteine	moderate	high					
Cathepsin G	*CTSG*	Serine	nil	nil					
Cathepsin H	*CTSH*	Cysteine	very low	nil	x				
Cathepsin K	*CTSK*	Cysteine	very low	nil					
Cathepsin L (L1)	*CTSL*	Cysteine	very low	nil	x	x	x		x
Cathepsin V (L2)	*CTSV*	Cysteine	very low	high					
Cathepsin O	*CTSO*	Cysteine	low	moderate					
Cathepsin S	*CTSS*	Cysteine	low	high	x		x		
Cathepsin W	*CTSW*	Cysteine	nil	nil					
Cathepsin Z (X)	*CTSZ*	Cysteine	low	moderate	x				
Reference	* *				[38]	[39]	[40]	[41]	[42]

*Cathepsins that were selected for silencing based on their moderate or high brain Human Protein Atlas protein levels are shaded in grey in this table.

**Fig 4:**
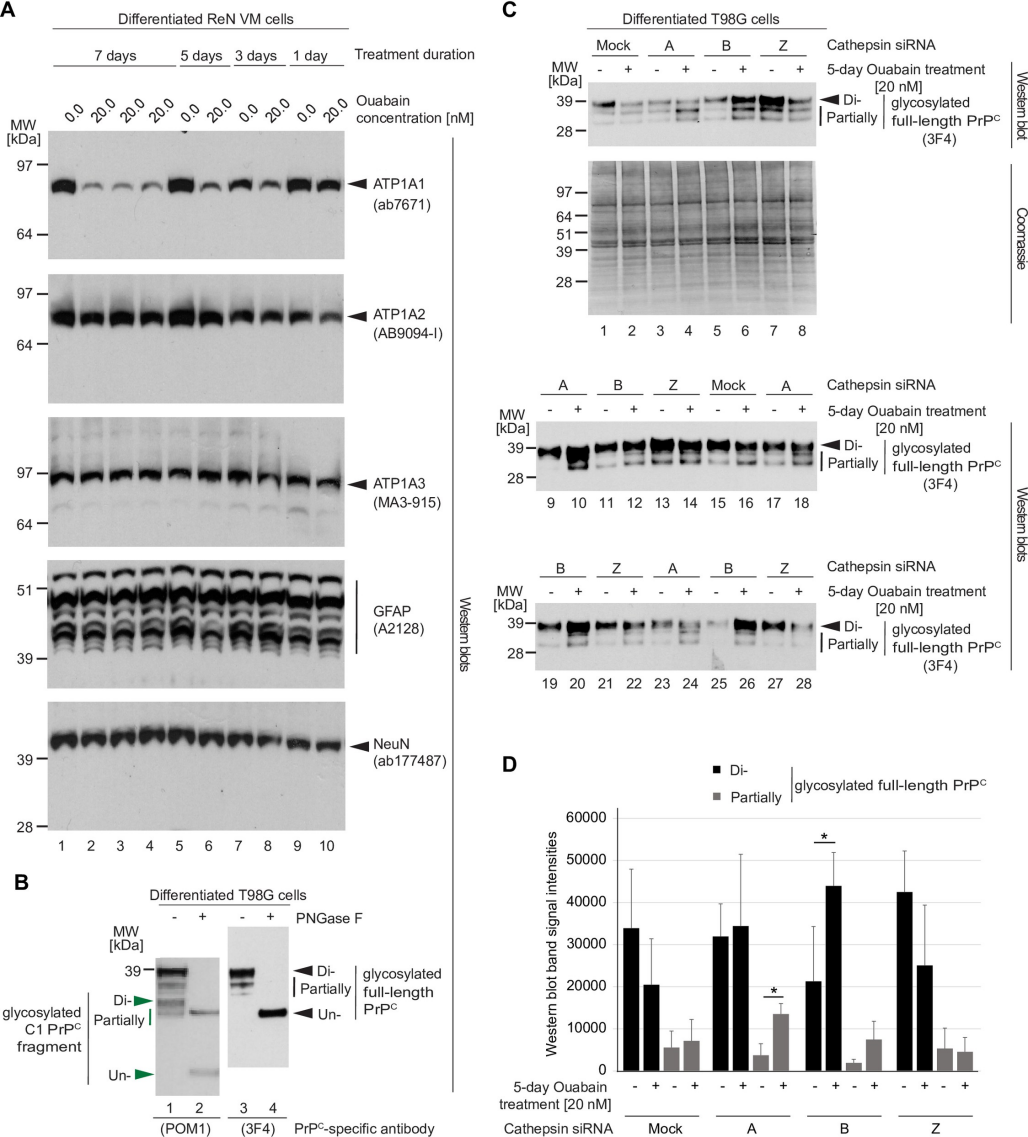
Cathepsin B is the main cathepsin involved in the ouabain-induced reduction in PrP^C^ levels. (A) Treatment with 25 nM ouabain over 3 to 7 days is sufficient to induce a profound reduction in ATP1A1 protein levels in T98G cells. (B) PrP^C^ in T98G cell extracts consists primarily of diglycosylated full-length PrP^C^, with partially N-glycosylated and endoproteolytically cleaved PrP^C^ representing a minor sub pool. (C) Western blot and Coomassie SDS-PAGE analyses of cell extracts from T98G cells silenced for individual cathepsins (as indicated) and treated with 25 nM ouabain or vehicle in the cell culture medium for a duration of four days. Probing of the western blot with the anti-human PrP antibody 3F4 led to three bands per lane, with the slowest migrating band representing diglycosylated full-length PrP^C^ and faster migrating bands representing partially N-glycosylated full-length PrP^C^. As expected, mock silencing did not prevent the ouabain-induced reduction in full-length PrP^C^ levels (compare Lanes 1 and 2). The same was observed after silencing of cells with a cathepsin Z-directed siRNA pool (compare Lanes 7 and 8). Although silencing of cathepsin A increased PrP^C^ levels of all sizes, it had only a minor effect on stabilizing levels of full-length PrP^C^ (compare Lanes 3 and 4). Finally, silencing of cathepsin B rescued the ouabain-dependent diminution of full-length PrP^C^ levels (compare Lanes 5 and 6). The amounts of total proteins analyzed was equal for all samples following adjustment by bicinchoninic acid (BCA) assay analysis. (D) Graph depicting results of densitometry analyses of western blot signals shown in Panel B. The analyses were undertaken separately for the slowest migrating 3F4-reactive bands, which were interpreted to represent N-glycosylated full-length PrP^C^ (black color), and for slower migrating 3F4-reactive PrP^C^ bands (grey color). Bars indicate the means of signal intensities and their standard deviations. Were indicated with asterisks, sample cohort comparisons met the significance threshold of *p*<0.05.

Next, we sought to determine the shortest ouabain treatment duration that leads to a pronounced reduction in ATP1A1 levels because we anticipated that it might be a challenge to transiently silence individual cathepsins for more than four days. To this end, we treated one-week differentiated ReN VM cells with vehicle or 20 nM ouabain for 1, 3, 5, and 7 days, then compared NKA alpha subunit levels in their extracts. This experiment revealed that ouabain treatments extending beyond three days are sufficient to achieve robust reductions in steady-state ATP1A1 protein levels (Fig 4A). To gain additional insights into whether ouabain exposure alters the astrocytic or neuronal differentiation following different durations of exposure, we also probed western blots with GFAP- and NeuN-directed antibodies, respectively. These analyses corroborated the conclusion that the balance of astrocytes and neurons does not change by the ouabain treatment during all treatment durations tested.

When subsequently optimizing the cathepsin silencing conditions, it came to the fore that T98G cells represent a better transfection host than ReN VM cells, which is why we moved to using T98G cells, an immortalized human glioblastoma multiforme tumor cell line, for cathepsin mRNA silencing experiments. As for ReN VM cells, PrP^C^ in the T98G cell model consists primarily of diglycosylated full-length PrP^C^ (Fig 4B). Next, we transfected T98G cells with validated pools of siRNAs with the intent to silence the expression of one of the cathepsins of interest per cell culture dish, then added 20 nM ouabain or vehicle solution to the cell culture medium. With this experimental design, if we silenced the expression of a cathepsin that plays a role in the ouabain-induced reduction in full-length PrP^C^ levels, we anticipated a rescue of this phenotype, thereby leading to similar PrP^C^ levels in cells exposed to ouabain versus vehicle solution. The western blot-based evaluation of this experiment revealed that the silencing of cathepsin B led to a profound rescue of the ouabain-induced degradation of full-length PrP^C^ (Fig 4C). The rescue of cathepsin A also led to an increase in PrP^C^ levels but this increase was most prominent for the faster migrating partially glycosylated PrP^C^ bands, as opposed to the slowest migrating diglycosylated full-length PrP^C^ that is most reduced upon CG exposure (Fig 4D). In contrast, the silencing of all other cathepsins tested, i.e., cathepsins D, F, L2 (V), S and X (Z) (not shown), did neither prevent the ouabain-induced reduction in diglycosylated full-length PrP^C^ levels nor lead to a significant increase in overall PrP^C^ levels. Taken together, these experiments pointed toward cathepsin B as the main endo/lysosomal cathepsin responsible for the CG-induced reduction in PrP^C^ levels.

### Comparison of potency of ouabain and oleandrin for reducing PrP^C^ levels

**Fig 5:**
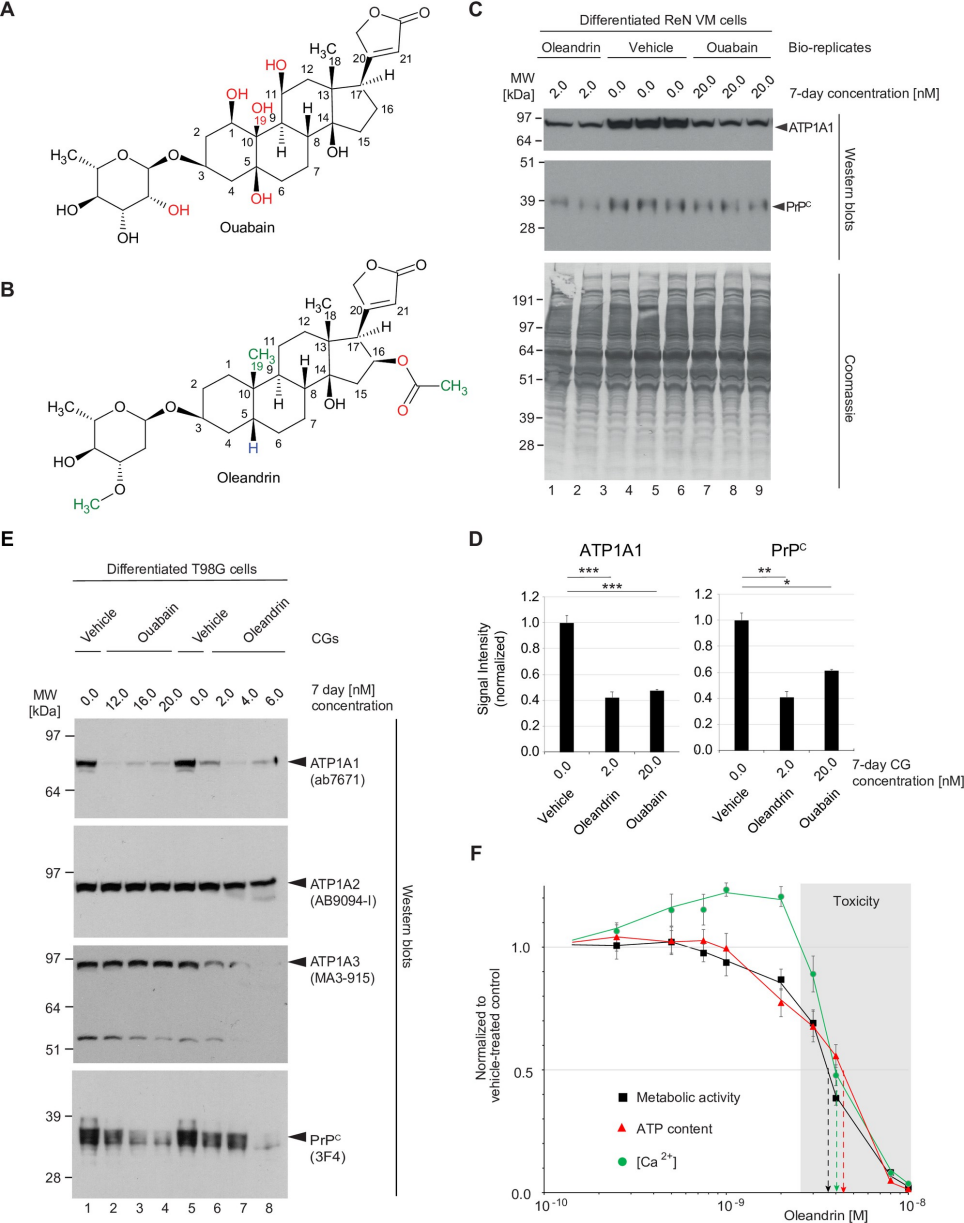
Oleandrin is a potent yet toxic CG for the NKA-mediated reduction of PrP^C^ levels. Comparison of (A) ouabain, a cardiotonic steroid sourced from the *Strophanthus gratus* plant native to eastern Africa, and (B) oleandrin, found in *Nerium oleander*, an ornamental shrub of uncertain origin. A lack of hydroxyl groups in the steroid core and the presence of additional methyl groups that are absent in ouabain account for the more hydrophobic characteristics of oleandrin. (C) Tenfold lower levels of oleandrin than ouabain cause a similar reduction in steady-state PrP^C^ levels during 7-day treatment of differentiated ReN VM cells. (D) Graphs comparing the means of western blot signal intensities of ATP1A1 and PrP^C^ levels and their standard deviations in 2 nM oleandrin- versus 20 nM ouabain-treated ReN VM cells. The number of asterisks shown reflect significance thresholds as follows: one: *p*<0.05; two: *p*<0.005; three: *p*<0.0005. (E) The potency of oleandrin translates to T98G cells and may reflect an ability of this CG to reduce not only levels of ATP1A1 but also ATP1A3. (F) Influence on cell viability (black squares), cellular ATP content (red triangles) and intracellular Ca^2+^ concentration ([Ca^2+^]_i_, green circles) of 7-day exposure of ReN VM cells to a range of oleandrin concentrations (10 pM to 10 nM). Toxic concentrations of oleandrin (exceeding 3 nM) are shaded in grey.

Because neither ouabain nor digoxin are suitable for brain applications due to their relative low membrane permeability [44] and active extrusion from the brain by P-glycoproteins [45–47], we next explored alternative CGs known to have higher blood brain barrier (BBB) penetrance. Amongst several CGs we considered was oleandrin, a compound that can be extracted from the ornamental plant *Nerium oleander*. This compound has lower hydrogen bonding capacity than ouabain due to the replacement of several hydroxyl groups on both its steroid core and glycoside, a feature predicted to improve its brain penetrance [48] (Fig 5A and 5B).

A side-by-side comparison of oleandrin and ouabain revealed an approximately tenfold higher potency of oleandrin toward differentiated ReN VM cells based on achieving a similar reduction in steady-state levels of ATP1A1 and PrP^C^ during a 7-day treatment regime (Fig 5C and 5D). Critically, this observation did not represent an idiosyncrasy of differentiated ReN VM cells, because differentiated T98G cells also responded to ouabain or oleandrin treatment with a concentration-dependent reduction in PrP^C^ levels. A closer investigation of how these CGs affected the levels of the three NKA alpha subunits in T98G cells corroborated the ReN VM cell analysis-based conclusion that ouabain mainly reduces ATP1A1 levels (see Fig 2A). In contrast, oleandrin affected both the levels of ATP1A1 and ATP1A3 profoundly (Fig 5E). Interestingly, the oleandrin concentration-dependent reductions in ATP1A3 levels seemed to correlate with reductions in cellular health. More specifically, exposure to oleandrin levels of up to 2 nM was well tolerated by differentiated ReN VM cells or T98G cells even over prolonged time periods exceeding one week based on their metabolic activity, cellular ATP and Ca^2+^ levels. Further increases in oleandrin concentrations beyond 3.5 nM caused these measures of cellular health to drop during a one-week treatment course to levels below 50% of values observed in mock-treated cells (Fig 5F).

### Influence of oleandrin on PrP^C^ levels in differentiated ReN VM cells depends on ATP1A1 engagement

**Fig 6:**
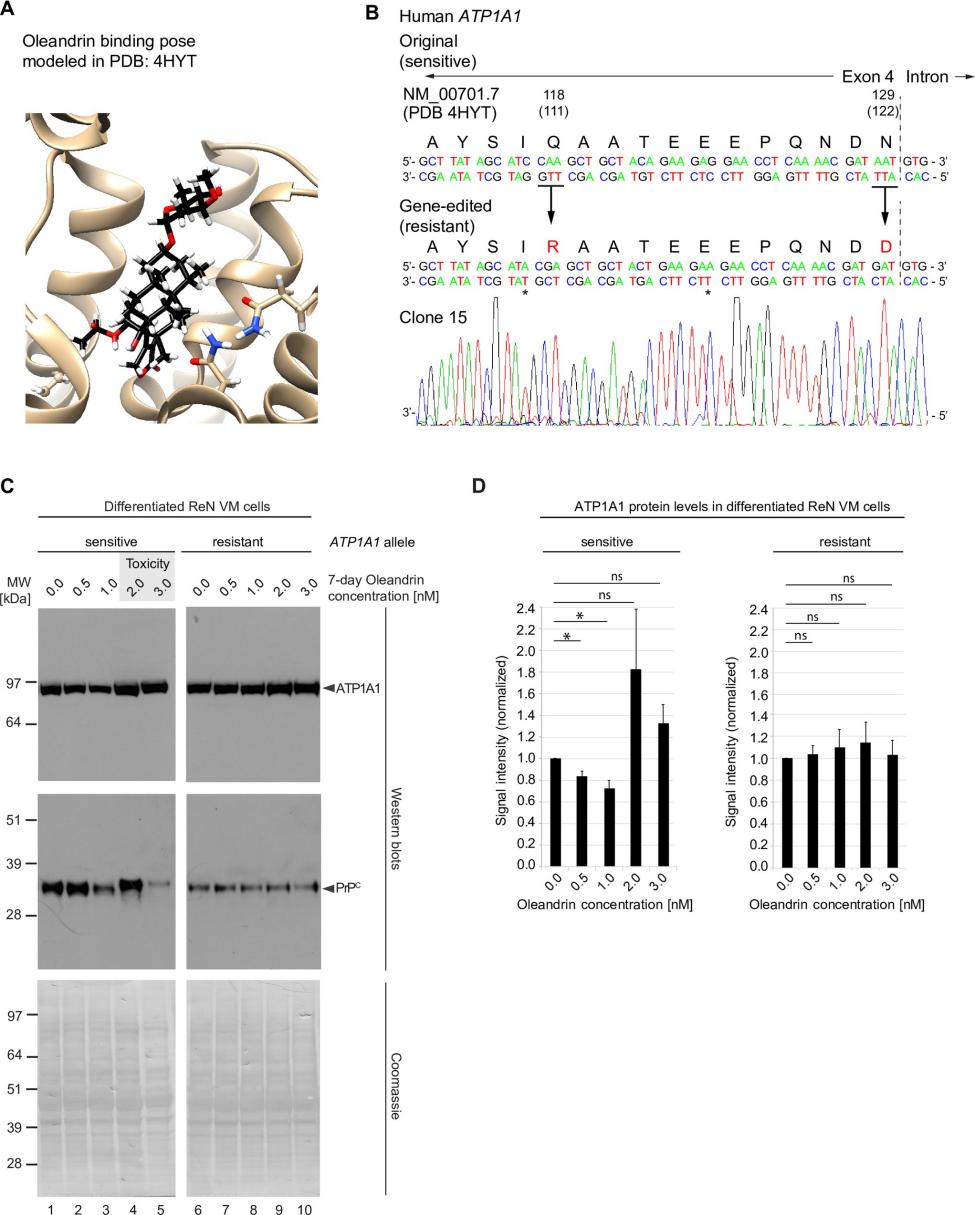
CG-dependent reduction of PrP^C^ levels depends on CG-ATP1A1 ligand-receptor engagement. (A) Predicted oleandrin binding pose modeled in ATP1A1 structure reported in PDB entry 4HYT. (B) Genetic sequencing results confirm successful amino acid substitutions Q118R and N129D, two changes required to render the human ATP1A1 resistant to CGs, in a ReN VM clone. (C) Comparison of ATP1A1 levels (top panel) and PrP^C^ levels (bottom panel) in response to oleandrin treatment in ReN VM cells that express either the CG sensitive wild-type or the CRISPR-Cas9-engineered CG resistant ATP1A1 alleles. In wild-type cells levels of ATP1A1 and PrP^C^ decreased as oleandrin concentration increased from 0 to 1 nM. When oleandrin was administered at a concentration of 2 nM, levels of ATP1A1 and PrP^C^ rebounded, and toxicity was observed (grey shaded box). In contrast, steady-state protein levels of ATP1A1 and PrP^C^ remained stable at all oleandrin concentrations tested in ReN VM cells expressing the resistant form of ATP1A1, and no toxicity was observed. Coomassie staining documents that protein loading was adjusted across lanes. (D) Graphs depicting quantitation of steady-state levels of ATP1A1 in the presence of oleandrin concentrations up to 3 nM in CG-sensitive wild-type and CG-resistant ReN VM cells.

Several scenarios could be invoked regarding the mechanism of action through which CGs impact PrP^C^ homeostasis in a manner that might promote its endo/lysosomal degradation, including the possibility that cholesterol-like features of molecules in this compound class can disrupt the cholesterol-rich raft membrane domains that PrP^C^ resides in [49]. The aforementioned experiments had already revealed a reality whereby the specific NKA α-subunit that is reduced depends on the specific CG utilized (Figs 1 and 5E). We therefore were leaning toward the hypothesis that the diminution of PrP^C^ steady-state levels relies on a direct engagement of CGs with their NKA receptor targets and the subsequent co-internalization of PrP^C^ with these CG-NKA receptor complexes. Because ouabain treatment only affects ATP1A1 yet profoundly reduced steady-state PrP^C^ levels, we reasoned that a reduction of this specific NKA α subunit may also be sufficient when cells are exposed to oleandrin, which was reducing the protein levels of both ATP1A1 and ATP1A3. Following this rational, we applied precise gene editing using CRISPR-Cas9 technology to engineer ReN VM cells that express a mutated CG-resistant derivative of the ATP1A1 isoform. The design of this gene editing step capitalized on prior work by others, which had shown that a glutamine (residue 111) and an asparagine (residue 122) residue in the CG binding pocket of the ATP1A1 are critical for CG binding (Fig 6A). Exchanging these two residues to arginine and aspartic acid, amino acids present in these positions in several rodent ATP1A1 orthologs, is known to lower the CG binding affinity of this isoform approximately 1000-fold [50, 51] and is the reason for dramatically higher tolerances of rodents toward CG exposure [52]. Using a paired Cas9 nickase strategy, ReN VM cells were transfected with gRNAs, which caused staggered cuts near the genomic ATP1A1 target site that were subsequently repaired using a 200-nucleotide-long single-stranded oligonucleotide (ssODN) repair template carrying the intended point mutations. Several ReN VM cell clones were obtained, which were sequence-validated to be homozygous for the mutated human CG-resistant ATP1A1 isoform (Fig 6B). Next, the response of wild-type CG-sensitive and mutated CG-resistant ReN VM cells to a range of low nanomolar oleandrin concentrations was compared. In three biological replicates of this experiment, a consistent response of wild-type ReN VM cells was observed, which was characterized by an initial decline of ATP1A1 levels at oleandrin concentrations of 0.5 and 1 nM, followed by a sharp increase in steady-state pump levels at around 2 nM, which tended to get weaker at higher, more toxic concentrations (Fig 6C and 6D)—the observant reader may notice that the precise oleandrin concentration that caused this uptick in ATP1A1 steady-state levels seemed a bit lower in this experiment than in the aforementioned analyses (Fig 5C), thereby corroborating the interpretation that the toxicity threshold for this compound may be nearer to the 2–3 nM oleandrin range that the metabolic cell health indicator assays had revealed (Fig 5F). When the same samples were probed with PrP^C^-directed antibodies, a similar trend was observed. However, rather than leading to a mere rebound of PrP^C^ steady-state levels at 2 and 3 nM oleandrin concentrations, the PrP^C^ signal also migrated reproducibly slower at these borderline toxic concentrations of this CG. Critically, in cells that express the mutant CG-resistant ATP1A1 protein, neither the changes in ATP1A1 nor in PrP^C^ levels were observed. Taken together, these data revealed the formation of a high-affinity CG ligand–ATP1A1 receptor complex to be indispensable for the mechanism of action through which oleandrin exposure of human co-cultures of neurons and astrocytes triggers the endo/lysosomal degradation of PrP^C^ that translates into reduced steady state levels of this protein.

## Discussion

**Fig 7:**
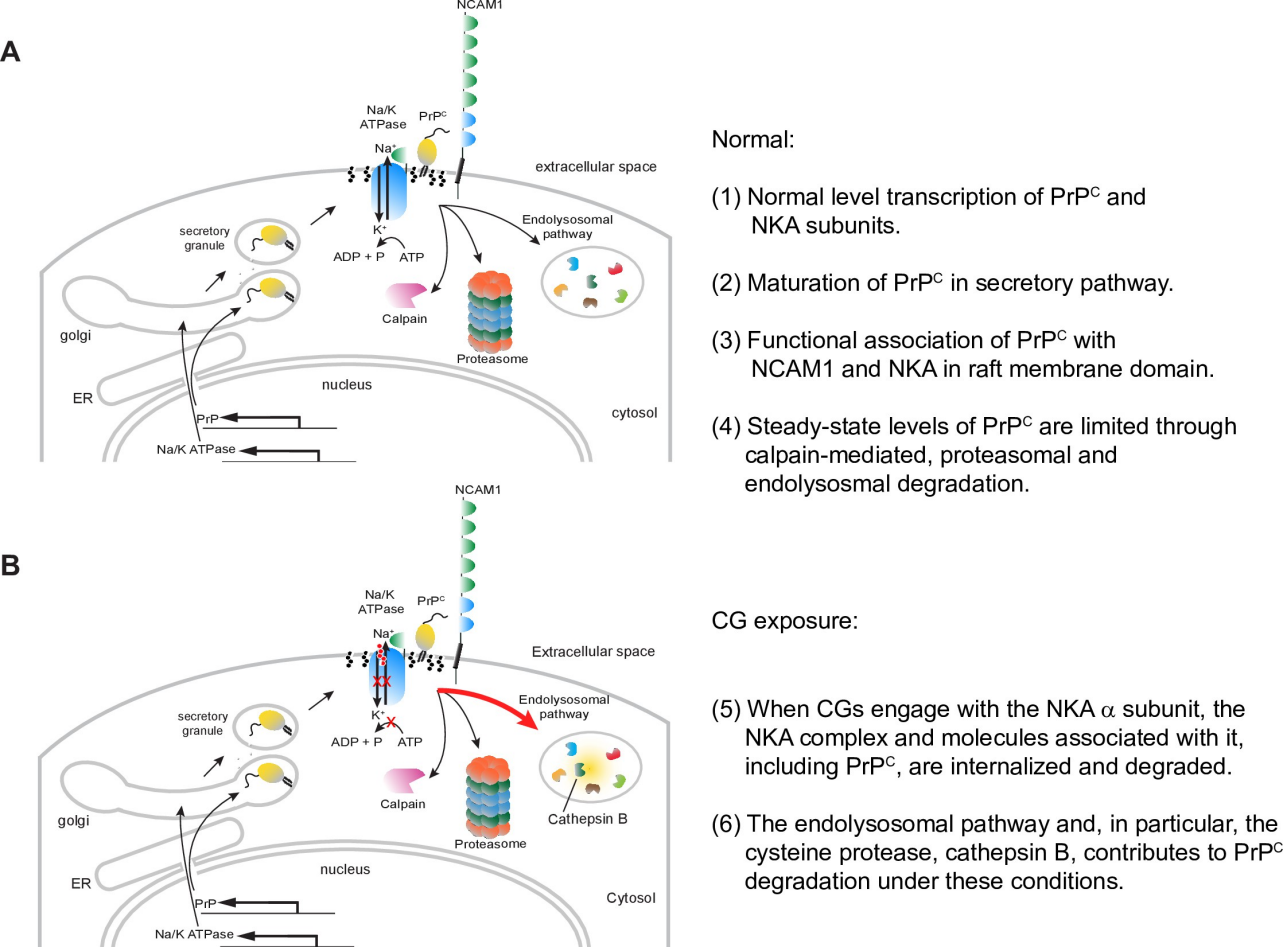
Model of CG-dependent reduction of PrP^C^. (A) Normal cell homeostasis. (B) Enhanced lysosomal degradation of PrP^C^ upon exposure to non-toxic levels of CGs.

This study established that steady-state PrP^C^ levels in human neural cell cultures can be reduced in a concentration-dependent manner by adding nanomolar levels of certain CGs to the cell culture medium. Initially working with ouabain and digoxin, we documented that the degree to which these CGs affect the levels of the three NKA α subunits known to be expressed in human brains is consistent with their previously reported NKA α subunit binding preferences. From these insights followed the observation that it is advantageous to select a CG that preferentially targets the ATP1A1 NKA α subunit if the goal is to reduce PrP^C^ levels in ReN VM cells and T98G cells, the specific neural cell models employed in this study. Notably, even the prolonged exposure of ReN VM cells with CGs did not alter the balance of astrocytic or neuronal markers in this co-culture model. We then observed oleandrin, a CG considered to have favorable brain bioavailability, to be highly potent but also rather toxic in this PrP^C^ reduction paradigm. Using CRISPR-Cas9-mediated mutagenesis of sites within the *ATP1A1* gene known to control CG binding affinity, we generated a ReN VM cell-derivative whose ATP1A1 α subunit is resistant to CG binding. Next, we showed that this ATP1A1 mutagenesis abolished the oleandrin-dependent reduction in the protein levels of both ATP1A1 and PrP^C^, thereby establishing the need for a direct engagement of the CG with its cognate ATP1A1 binding site for the protein level changes to occur. Finally, we provided evidence in support of the conclusion that the CG-dependent reduction in PrP^C^ levels depends on its endo/lysosomal degradation and can be rescued by silencing the expression of cathepsin B (Fig 7).

Although control Coomassie stains established that the steady-state levels of many cellular proteins were not affected when cells were exposed to CGs, PrP^C^ is unlikely to be the only protein, aside from NKAs comprising ATP1A1, that exhibited reduced steady-state levels in the presence of CGs. A case in point is CD109, which we had previously observed to reside in spatial proximity to PrP^C^ [15], whose steady-state levels were also reduced in CG exposed cells. Therefore, it seems reasonable to assume that upon CG engagement patches of membrane, most likely lipid rafts or caveolae, that harbor the targeted NKA, along with their closest molecular neighbors, are internalized by the cell. Sub pools of NKAs have increasingly been shown to associate with rafts [53, 54] and caveolae [55], a localization that might be facilitated by both their β subunits [56] and caveolin-binding domains within the their α subunits [57]. That this localization is not a niche observation can be deduced from robust data that 75% of total myocyte NKA activity was associated with caveolae-like membrane preparations [58] and NKAs within neurites map to hot spots interpreted as raft domains in neurites of cerebellar granule cells [53]. Once internalized, individual proteins within such a patch of membrane may experience distinct fates. So far, our study pointed at the endolysosomal pathway as the main system for degrading PrP^C^ in response to CG exposure. Additional mechanisms can be invoked that may contribute to the reduction in PrP^C^ levels but have not yet been tested, including the possible release through extracellular vesicles or posttranslational events that may escape detection with PrP-directed antibodies used in this study.

The study of NKAs is fraught with complexities: Their investigation in human and rodent cells has to account for the existence of four α and three β subunit paralogs that make up the main building blocks of these heteromeric protein complexes [59] (a fourth β subunit, ATP1B4, has in vertebrates acquired a novel function in the inner nuclear membrane [60]). Additional combinatorial complexity derives from the observation that individual α and β isoforms may exhibit preferential associations [61, 62] rather than fixed pairings [63]. Moreover, individual cells, including the neurons and astrocytes of the human ReN VM cell paradigm employed in this study, have distinct expression profiles of α subunits [64–66] and these profiles shift as the cells differentiate [67]. These complexities are raised here to make the point that there is still much to be learned regarding the intricacies of the molecular underpinnings through which CG exposure leads to a reduction in PrP^C^ levels.

Our results corroborate a growing body of evidence that points toward the existence of collaboration and built-in redundancies amongst NKA subunit paralogs. Consistent with this interpretation, the CG-mediated reduction in ATP1A1 levels was paralleled by a reduction also of ATP1A2 and an increase in ATP1A3 at slightly higher CG concentrations. The biological significance of shifting the relative abundances of the subunits remains unclear at this time. We also observed that the increase in CG concentrations toward borderline toxic levels generated a rebound in the levels of ATP1A1. One plausible explanation for this observation is the existence of a cellular response that senses and compensates for the pronounced diminution of NKA activity that manifests in the presence of higher CG levels by increasing the production and/or slowing the degradation of NKA subunits.

Because of its potential therapeutic implications, the most intriguing finding in this study was the observation that PrP^C^ levels in a human co-culture model of neurons and astrocytes can be reduced by more than 50% when cells are exposed to non-toxic levels of CGs, a class of compounds that is well-characterized in its clinical use following decades of research and use in patients with heart disease. Note that the mechanism through which CGs cause this diminution of steady-state PrP^C^ levels appears unrelated to the way another aminoglycoside, G418 (geneticin), impairs the *de novo* infection of cells with prions in the absence of an influence on PrP^C^ levels or its localization [68].

Several members of the CG compound class are well-known poisons to a wide range of species due to their exquisite potency and their targeting of a critical pump held responsible for maintaining the electrochemical gradient in eukaryotic cells. The upside of this potency is that CGs can achieve reductions in PrP^C^ levels at low nanomolar concentrations. This potency stands in contrast to previous data from a high-throughput screen of 1,280 compounds that were derived from the US Drug Collection and reported micromolar effective concentrations for the most favorable compounds, Tacrolimus and Acetimole [12]. Another systematic screen of a larger library of 44,578 compounds led to the identification of 32 small molecules that reduced PrP^C^ levels at 65 nM to 4.1 μM concentrations without killing the cells [11]. We suspect that initial cell survival screens undertaken in these two referenced high throughput studies would have eliminated CGs due to their exquisite potency and toxicity at levels exceeding micromolar concentrations.

The best-understood FDA-approved CGs have a narrow therapeutic window and reach lower concentrations in the brain than in peripheral tissue, features which are acceptable for the treatment of heart defects but a hindrance for possible neurodegenerative indications. However, there are reasons to think that these limitations are not insurmountable as we understand more about the constraints that limit access of CGs to the brain. For example, it is apparent that a subset of CGs are actively removed from the brain through the P-glycoprotein (P-gp) [69], which offers an opportunity to concomitantly restrict the activity of this antiporter system. In fact, studies in mice have established that brain levels of digoxin can be more than tenfold augmented in the presence of the P-gp inhibitor elacridar [46], or upon knockout of two P-gp genes conferring multidrug resistance (*mdr1a* and *mdr1b*) in mice [70]. Moreover, a subset of CGs, including the oleandrin compound found in milkweed plants [71] or neriifolin [72] have been reported to reach naturally higher levels in the brain than in peripheral tissues. More specifically, tritiated oleandrin has been observed to reach 83 nM brain levels 5 days after daily intravenous injections into cats [73] and 226 nM brain levels 24 hours after being intraperitoneally administered to mice [74].

Would it be safe to reduce NKA levels in the brain? Complete deficiency of any of the three NKA α subunits expressed in the brain is lethal *in utero* [75] or at birth [76, 77]. Moreover, mutations in NKA α subunit genes can cause severe phenotypes, including rapid-onset dystonia parkinsonism [78, 79], aldosterone-producing adenomas [80] and Charcot-Marie-Tooth neuropathy [81]. Critically, when encountered, these phenotypes have been interpreted as autosomal dominant gain-of-toxic function effects [82]. In contrast, available evidence, including from genetic studies, indicates that a partial reduction of NKA α subunit levels may be acceptable. More specifically, mice that were engineered to be heterozygous for any of the NKA phenotypes were reported to be viable and to exhibited moderate behavioral phenotypes but no visible neurological defects, such as tremors or seizures [77]. Similarly, phenotypes in humans associated with NKA α subunit heterozygosity tend to be less than severe, with forms of familial hemiplegic migraine being the most notable [83]. Consequently, when looking for a CG with favorable characteristics for the treatment of a brain disorder, key objectives would be to achieve low bioavailability in the heart, free concentrations in the brain that exceed levels required for NKA target engagement, and to select a CG that does not suppress the steady-state protein levels of any of the predominant NKA α subunits by more than 50%. Our previous observation that PrP^C^ resides in proximity to all three brain NKA α subunits in the brain [17] suggests to us that it might be possible to achieve a PrP^C^ brain level reduction of more than 50% with a CG that engages with the three brain NKA α subunits in a relatively balanced manner.

## Conclusions

Our study uncovered a novel therapeutic strategy for reducing steady-state levels of PrP^C^. The indirect targeting approach we proposed may also be applicable to other cell surface proteins for which attempts to identify compounds that bind to them directly have not been successful. To assess the clinical suitability of this strategy for removing cell-surface PrP^C^ necessitates future work that evaluates and optimizes CGs for their safe use in brain applications. The fact that this treatment strategy emerged from mechanism-based investigations—as opposed to a high-throughput compound screen—validates the power of basic omics-based discovery research and should facilitate the bench-to-bedside translation. Once a CG derivative with favorable pharmacological characteristics has been identified, a pre-clinical evaluation of the ability to extend survival in a suitable prion-infected animal model will be urgently needed. An important consideration for such a study poses the small differences in human versus rodent ATP1A1 sequences, which render NKAs of widely used rodent models comprising this subunit approximately a thousand-fold less sensitive to CG inhibition than their human orthologs.

## Materials and methods

### Antibodies

Primary antibodies were sourced as follows: mouse monoclonal (clone 3F4) anti-human PrP antibody (catalog number MAB1562, MilliporeSigma, Burlington, Ontario; used at 1:1000 dilution), mouse monoclonal (clone POM1) anti-PrP antibody (catalog number MABN2285, MilliporeSigma; used at 1:1000), mouse monoclonal IgG1 (clone 464.6) anti-ATP1A1 antibody reactive to various species (catalog number ab7671, Abcam, Waltham, MA; used at 1:2000 dilution), mouse monoclonal IgG1 (clone C-9) anti-CD109 reactive toward mouse, rat and human (catalog number sc-271085, Santa Cruz Biotechnology, Dallas, TX; used at 1:100 dilution), mouse monoclonal IgG1 (clone C4) anti-β-actin antibody reactive against a wide range of species (catalog number sc-47778, Santa Cruz Biotechnology; used at 1:1000 dilution). Horseradish peroxidase-conjugated secondary antibodies against mouse (catalog number 7076S; used at 1:5,000 dilution) and rabbit (catalog number 7074S; used at 1:5000 dilution) immunoglobulin G were from Cell Signaling Technology and distributed by New England Biolabs, Whitby, ON.

### Cardiac glycosides

Ouabain octahydrate (catalog number O3125, Sigma-Aldrich, Oakville, ON), digoxin (catalog number D6003, Sigma-Aldrich), and oleandrin (catalog number 06069, Sigma-Aldrich) were initially dissolved in water or dimethylsulfoxide (DMSO), and further diluted in cell culture media to 1000 x working concentrations prior to treatments. For treatments of differentiated ReN VM cells spanning several days, CG levels were replenished daily along with a change of half of the cell culture medium.

### Cell culture

ReN VM cells were maintained in DMEM/F12 (catalog number 11320033, Thermo Fisher Scientific) supplemented with 2% N21-MAX (catalog number AR008, R & D Systems, Minneapolis, MN) or 2% B27(catalog number 17504044, Thermo Fisher Scientific), 20 ng/mL basic fibroblast growth factor (catalog number PHG0261, Thermo Fisher Scientific), 200 ng/mL epidermal growth factor (catalog number RKP01133, Reprokine, Tampa, FL), and 2 ng/mL heparin (catalog number H3149-10KU, Sigma-Aldrich) on Matrigel-coated (catalog number 354230, Corning, Guelph, ON) tissue culture plates at 37°C with 5% CO_2_ as previously described [84]. ReN VM cells were differentiated into co-cultures of neurons and astrocytes upon removal of growth factors and heparin for at least 7 days, as previously described [18]. For cryogenic preservation of cell stocks in liquid nitrogen, cells were stored in Recovery Cell Culture Freezing Medium (catalog number 12648010, Thermo Fisher Scientific).

T98G cells were maintained in DMEM (catalog number 11965092, Thermo Fisher Scientific) supplemented with 10% fetal bovine serum (catalog number 12483020, Thermo Fisher Scientific), 1% Glutamax (catalog number 35050061, Thermo Fisher Scientific) and 50 U/mL Penicillin + 50 ug/mL Streptomycin (catalog number 15070063, Thermo Fisher Scientific). One day prior to CG treatment and for the duration of the treatment course, T98G cells were maintained in serum-free media to halt proliferation. Both ReN VM and T98G cells were maintained at 37°C and 5% CO2 in a humidified incubator.

### Immunocytochemistry and confocal microscopy

ReN VM cells were cultured as described above. Cells were fixed in 4% formaldehyde for 15 minutes and permeabilized with 0.1% Triton X-100 for 20 minutes. Permeabilized cells were incubated overnight at 4°C in PBS buffer containing 1% BSA and primary antibody directed against ATP1A1 (catalog number ab7671, Abcam, Waltham, MA; used at 1:200). Following three PBS washes, Alexa-Fluor 633 secondary antibody (catalog number A-21052, Invitrogen, Burlington, ON; used at 1:400) was incubated with cells for 90 minutes at ambient temperature in PBS buffer containing 1% BSA. Cells were washed three times in PBS and mounted onto glass slides using ProLong Gold containing DAPI (catalog number P36934, Invitrogen, Burlington, ON). Z-stack images were captured using an LSM880 inverted confocal microscope (Carl Zeiss Canada Ltd, Toronto, ON) and processed using Zen Blue software (Carl Zeiss Canada Ltd).

### Effects of digoxin and oleandrin on cell viability, intracellular Ca^2+^, and ATP content

ReN VM cells were plated at 16,800 cells/well on a Matrigel-coated black 96-well clear bottom tissue-culture plate. Cells were grown in proliferation media for two days before being differentiated by growth factor withdrawal for one week. After differentiation, cells were treated with digoxin or oleandrin. After one week of treatment, cells were analyzed with the following assays:

*Cell viability assay using calcein-AM* (catalog number 17783, Sigma-Aldrich). Cells were washed twice with PBS (catalog number D8537-500ML, Sigma-Aldrich) and then incubated with 1 μM of calcein-AM in PBS with 3% (w/v) bovine serum albumin (BSA) (catalog number ALB001.50, BioShop, Burlington, ON) for 20 min at 37°C. Cells were then rinsed with phosphate buffered saline (PBS) to remove calcein-AM before being incubated for another 30 min at 37°C in just PBS with 3% (w/v) BSA to allow complete de-esterification of AM esters. Excitation/emission signal at 486/516 nm was measured in a microplate reader as an indication of cell viability.

*Cellular ATP content assay using CellTiter-Glo* (catalog number G7570, Promega, Madison, WI). Cells were equilibrated to room temperature for 30 min to minimize uneven plate reading due to inconsistent temperature. The CellTiter-Glo reagent was diluted 1:4 in PBS before use. The volume of diluted CellTiter-Glo reagent added per well was equal to the volume of differentiation media already present in each well. Plates were then shaken for 2 min on an orbital shaker for 2 min at room temperature to induce cell lysis. Luminescence was recorded with an integration time of 1 s as an indication of cellular ATP content.

*Intracellular Ca*^*2+*^ *content assay with Fluo-4 AM* (catalog number F14201, Thermo Fisher Scientific). Cells were washed twice with PBS and then incubated with 2 μM of Fluo-4 AM in PBS with 3% (w/v) BSA for 20 min at 37°C. Cells were then rinsed with PBS to remove Fluo-4 AM before being incubated for another 30 min at 37°C in just PBS with 3% (w/v) BSA to allow complete de-esterification of AM esters. Excitation/emission signal at 486/516 nm was measured in a microplate reader as an indication of intracellular Ca^2+^ content.

### CRISPR-Cas9 mediated mutagenesis of human *ATP1A1* rendering resistance to CG exposure

ReN VM cells expressing the CG resistant form of *ATP1A1* were generated by transfection using a paired Cas9 nickase design based on two sgRNAs with the protospacer sequences ‘gttcctcttctgtagcagct’ and ‘gagttctgtaattcagcata’, supplemented with a 200-nt-long single-stranded oligonucleotide (ssODN) repair template (Ultramer Oligos, Eurofin Genomics, Toronto, ON). The sgRNAs were designed using the CHOPCHOP CRISPR design tool (http://chopchop.cbu.uib.no/) and selected based on high specificity scores and proximity to the target amino acid residues 118 and 129. The protospacer sequences were inserted into the MLM3636 sgRNA plasmid via site-directed mutagenesis using the Q5 Site-Directed Mutagenesis kit (catalog number E0554S, New England Biolabs, Ipswich, MA). The Cas9 D10A nickase plasmid was generated by site-directed mutagenesis as previously described [85]. All plasmids were transformed into 5- Competent *E*. *coli* (catalog number C2987H, New England Biolabs). All plasmids were purified using the PureLink HiPure Plasmid Filter Maxiprep Kit (catalog number K2100-16, Thermo Fisher Scientific) before transfection.

#### Transfection and selection

ReN VM cells were detached with Accutase (catalog number A1110501, Invitrogen, Burlington, ON) and plated at 135,000 cells/well in Matrigel-coated 12-well plates one day before transfection in proliferation media without heparin. Cells were then transfected at a ratio of 500 ng total DNA/well: 1 μL/well of TransfeX transfection reagent (catalog number ACS-4005, American Tissue Culture, Gaithersburg, MD). The ratio of plasmids used was 6 Cas9 nickase: 1 sgRNA: 1 sgRNA: 5 ssODN. 48 h post transfection, cells were treated with 100 nM of ouabain for 7 days to select for cells that have been edited to express the resistant form of *ATP1A1*. Surviving clones were picked for genomic analysis and cryopreservation.

#### Genomic PCR

Genomic DNA was extracted from clones grown on 12-well plates using the PureLink Genomic Mini Kit (catalog number K182001, Invitrogen). Precise transgene insertion into the target site at the *ATP1A1* locus was determined by genomic PCR using 25 ng of genomic DNA amplified for 28 cycles using ‘tttgtcggcagctctttggg’ and ‘agtgggagacaaagacggaga’ as the forward and reverse primers, respectively. PCR products were purified with a gel/PCR extraction kit (catalog number DF300, Froggabio, ON).

### *In vitro* protease inhibition studies in ReN VM cells

Inhibitors were added two hours before the addition of the CGs. Calpastatin (7316, Clontech, CA, USA), Calpain inhibitor I (A6185, Sigma-Aldrich), MG132 (M7449, Sigma-Aldrich), Lactacystin (L6785, Sigma-Aldrich), Bafilomycin A1 (SML1661, Sigma-Aldrich), Pepstatin A (P5318, Sigma-Aldrich), E64D (E8640, Sigma-Aldrich) were dissolved in DMSO in 1000x stocks. Unless specified otherwise, MG132, E64D and bafilomycin A1 treatments were performed for 24hrs, calpain inhibitor I for three days and calpastatin for five consecutive days with daily replenishment.

### Protein extraction and immunoblotting

Cells were harvested in lysis buffer containing 1% NP40, 150mM NaCl and 150mM Tris-HCl (pH 8.3) supplemented with cOmplete protease and phosphatase inhibitors (catalog numbers 11836170001 and 4906837001; MilliporeSigma) and subjected to immunoblotting. Briefly, total protein amounts were adjusted across samples using a bicinchoninic assay (BCA) kit (23225, Thermo Fisher Scientific). Equal amounts of total proteins in extract samples were run on SDS-PAGE gels and transferred to 0.45 μm polyvinylidene fluoride (PVDF) membranes (IPVH00010, MilliporeSigma). Unspecific binding sites in PVDF membranes were blocked with 10% skim milk, followed by overnight incubation with primary antibodies. After extensive washing with TBS-Tween, the membranes were incubated with secondary antibodies and immunoreactive bands were visualized on X-ray films or a LI-COR Odyssey Fc digital imaging system (LI-COR Biosciences, NE).

### Removal of N-glycans by PNGase F digestion

For the removal of N-glycans from PrP^c^, PNGase F digestion was performed according to the manufacturer’s protocol (catalog number P0704S, New England Biolabs). Briefly, equal amounts of protein were denatured using 10 x Denaturing Buffer at 95°C for 10 minutes. Next 10 x NP40 and 10 x GlycoBuffer and PNGase F enzyme were added to the denatured samples. Samples were incubated overnight at 37°C. The next day, an equal volume of 2 X LDS sample buffer was added to the samples and proteins were separated on an SDS-PAGE gel and subject to immunoblotting.

### siRNA-based knockdown of endo/lysosomal proteases

T98G cells were plated at 30% confluency in 6-well plates and allowed to settle for 24 hrs. The growth medium was changed to serum-free medium when the cells reached 50% confluency. The next day, the cells were subjected to Lipofectamine RNAiMax (catalog number 13778040, Thermo Fisher Scientific) transfection reagent only or were transfected with validated pools of siRNAs (Silencer Select, Thermo Fisher Scientific) targeting the human cathepsins of interest (see **Table 1**) at a working concentration of 90 nM Silencer Select siRNA in Opti-MEM medium (Thermo Fisher Scientific). One day after the transfection, the cells were exposed to vehicle solution or 20 nM ouabain for a treatment that lasted a total of five days. Every other day, half of the cell medium was replaced with fresh prewarmed medium comprising again vehicle solution or 20 nM ouabain. The cell harvest and subsequent western blot analyses proceeded as described in the ‘Protein extraction and immunoblotting’ section.

### Statistical analyses

The three assays applied to evaluate cellular health were based on six biological replicates per concentration tested with randomized well assignment for each replicate to reduce bias caused by evaporation effect. The siRNA-based silencing experiments were also based on four biological replicates. Other western blot-based comparisons of signal intensities made use of three biological replicates. The statistical analyses of all cohorts assumed that the variance of grouped samples was unknown and, consequently, made use of the two-tailed t-test. Consistent with conventions in biomedical research, a p-value of less than 0.05 was considered significant and is indicated with a single asterisk. One additional asterisk is shown for every tenfold lower *p*-value that a given t-test returned. If t-tests were undertaken but the p-values they returned did not meet the significance threshold, this is indicated with the abbreviation ‘ns’.

### Ethics statement

This study made use of human immortalized cell lines. The use of these cell lines for the applications detailed in this report was reviewed and authorized (Biosafety Permit 208-S06-2) by the Environmental Health a Safety office at the University of Toronto, Toronto, Ontario, Canada.

## Supporting information

S1 Raw images Original images. Western blots and Coomassie-stained gel images. Note that several western blots were cut horizontally or vertically prior to detection with antibodies targeting proteins of distinct apparent molecular weights. Lanes that were either used to separate molecular weight markers or samples not used for this manuscript are indicated with M and X labels, respectively.
(PDF)
