# Supplementary material for: Cardiac glycoside-mediated turnover of Na, K-ATPases as a rational approach to reducing cell surface levels of the cellular prion protein
Source: PLoS One. 2022 Jul 1;17(7):e0270915. doi: 10.1371/journal.pone.0270915 (PMC9249225; doi:10.1371/journal.pone.0270915)

S1 raw images

Original images (figure and panel numbers shown in this document reflect those in the main manuscript, and marker (M) or unused (X) lanes are indicated)

Figure 1

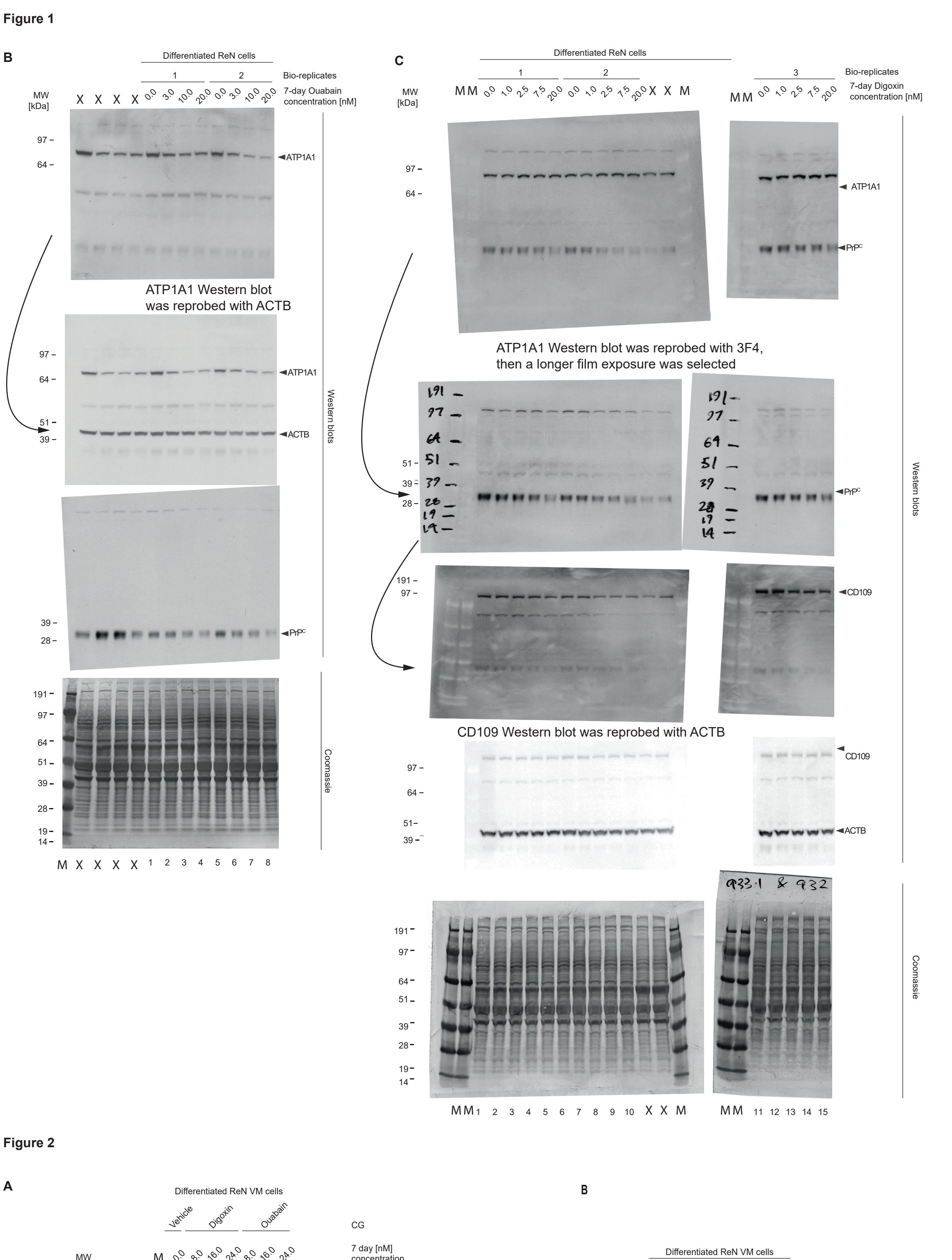

Figure 2

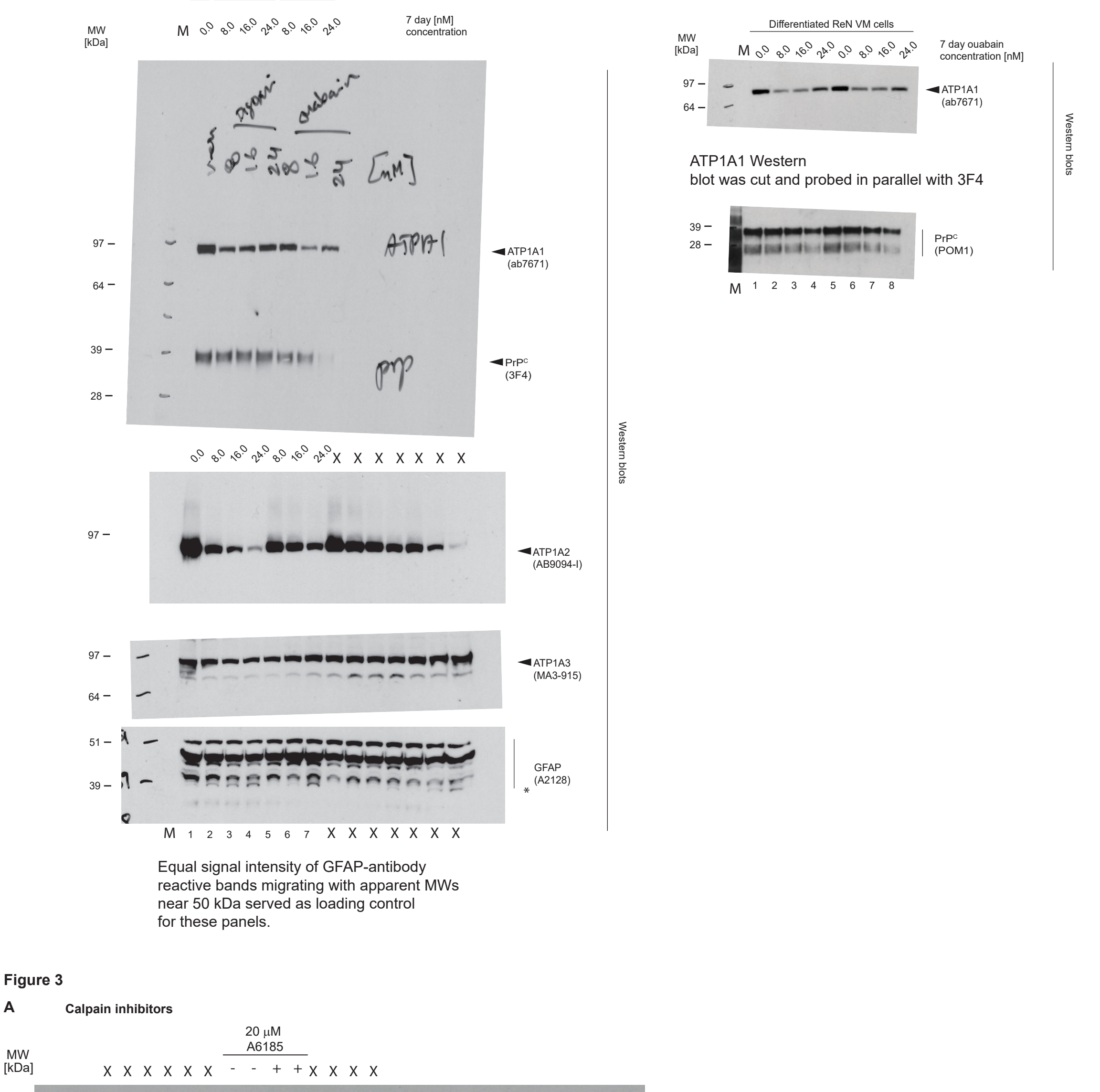

Figure 3

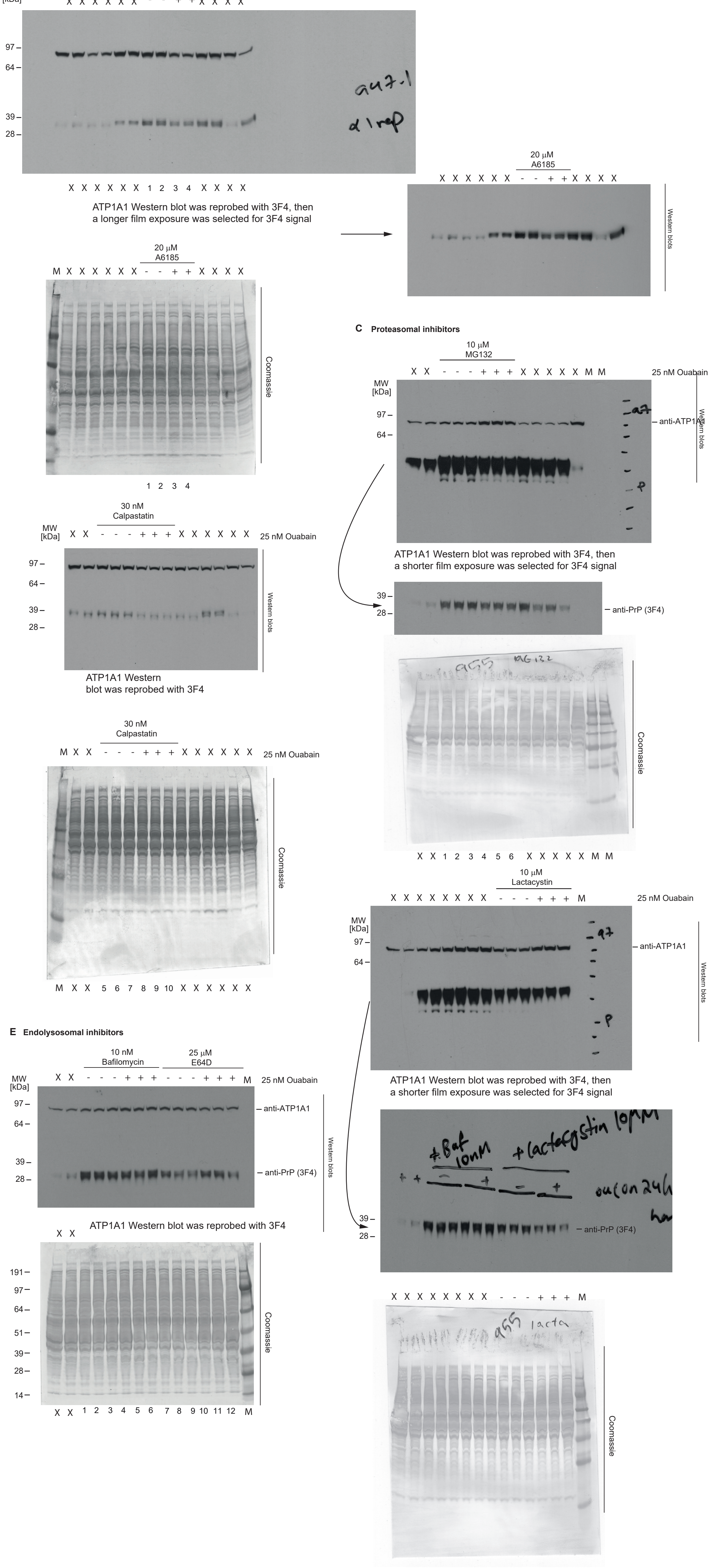

Figure 4

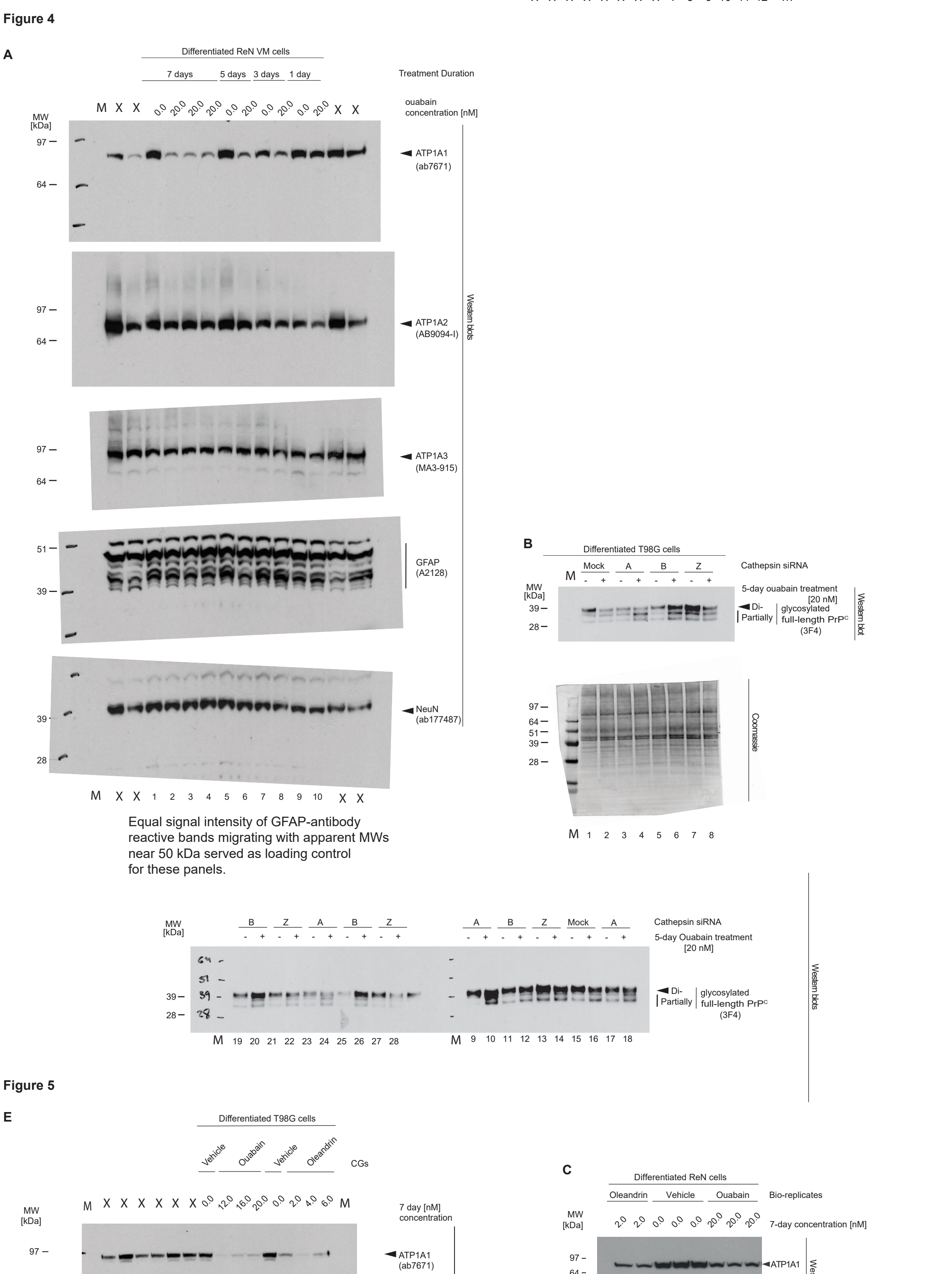

Figure 5

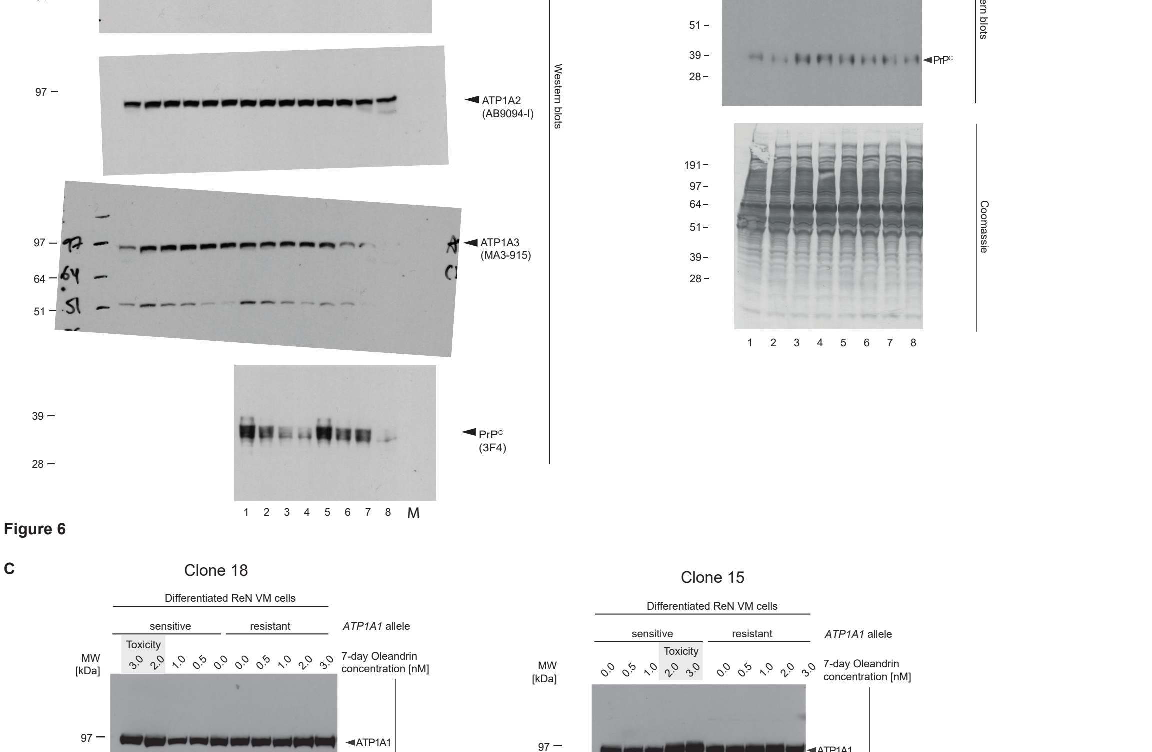

Figure 6

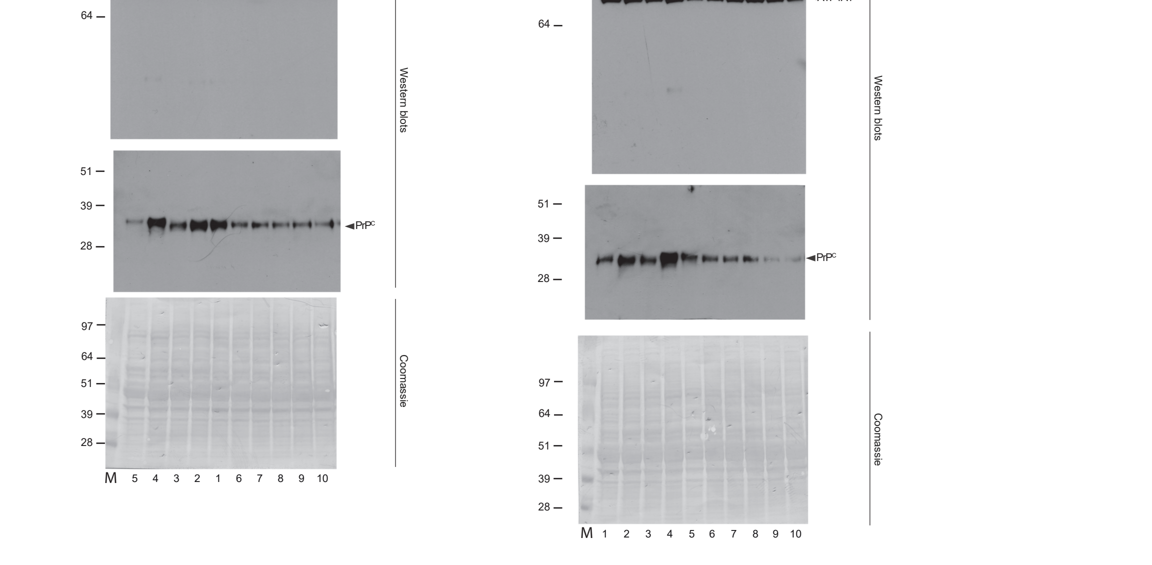

Supplement: S1 Raw images — Western blots and Coomassie-stained gel images. Note that several western blots were cut horizontally or vertically prior to detection with antibodies targeting proteins of distinct apparent molecular weights. Lanes that were either used to separate molecular weight markers or samples not used for this manuscript are indicated with M and X labels, respectively. (PDF) [file pone.0270915.s001.pdf]
